# MicroRNA-20a Constrains p300-Driven Myocardial Angiogenic Transcription by Direct Targeting of p300

**DOI:** 10.1371/journal.pone.0079133

**Published:** 2013-11-13

**Authors:** Lina A. Shehadeh, Salil Sharma, Mônica Pessanha, Jian Qin Wei, Jing Liu, Huijun Yuan, Claudia O. Rodrigues, Michaela Scherr, Nicholas F. Tsinoremas, Nanette H. Bishopric

**Affiliations:** 1 Department of Medicine, Division of Cardiology, University of Miami Miller School of Medicine, Miami, Florida, United States of America; 2 Departments of Molecular and Cellular Pharmacology, University of Miami Miller School of Medicine, Miami, Florida, United States of America; 3 Department of Pediatrics, University of Miami Miller School of Medicine, Miami, Florida, United States of America; 4 Center for Computational Sciences, University of Miami Miller School of Medicine, Miami, Florida, United States of America; 5 Department of Hematology, Hemostasis and Oncology, Hannover Medical School, Hannover, Germany; Brigham and Women’s Hospital, United States of America

## Abstract

**Objective:**

To characterize downstream effectors of p300 acetyltransferase in the myocardium.

**Background:**

Acetyltransferase p300 is a central driver of the hypertrophic response to increased workload, but its biological targets and downstream effectors are incompletely known.

**Methods and Results:**

Mice expressing a myocyte-restricted transgene encoding acetyltransferase p300, previously shown to develop spontaneous hypertrophy, were observed to undergo robust compensatory blood vessel growth together with increased angiogenic gene expression. Chromatin immunoprecipitation demonstrated binding of p300 to the enhancers of the angiogenic regulators Angpt1 and Egln3. Interestingly, p300 overexpression *in vivo* was also associated with relative upregulation of several members of the anti-angiogenic miR-17∼92 cluster *in vivo*. Confirming this finding, both miR-17-3p and miR-20a were upregulated in neonatal rat ventricular myocytes following adenoviral transduction of p300. Relative expression of most members of the 17∼92 cluster was similar in all 4 cardiac chambers and in other organs, however, significant downregulation of miR-17-3p and miR-20a occurred between 1 and 8 months of age in both wt and tg mice. The decline in expression of these microRNAs was associated with increased expression of VEGFA, a validated miR-20a target. In addition, miR-20a was demonstrated to directly repress p300 expression through a consensus binding site in the p300 3′UTR. *In vivo* transduction of p300 resulted in repression both of p300 and of p300-induced angiogenic transcripts.

**Conclusion:**

p300 drives an angiogenic transcription program during hypertrophy that is fine-tuned in part through direct repression of p300 by miR-20a.

## Introduction

In the normal heart, myocardial mass, vascular density and workload are closely balanced [1], [2], [3], [4]. Numerous studies have shown that this balance is perturbed in many experimental models of cardiac hypertrophy, and in clinical hypertension and aortic stenosis [5], [6], [7], [8]. In pressure-overload hypertrophy, capillary density fails to increase in proportion to cardiac mass, with resulting compromise in delivery of oxygen and nutrients. Hypertrophy appears to be associated with a specific defect in compensatory angiogenesis despite the presence of tissue hypoxia [9] and this deficit has been directly implicated in the conversion of compensatory to pathological hypertrophy in animal models, associated with impaired expression of angiogenic factors such as HIF-1α, HIF-2α and VEGFR2 [7], [9], [10], [11], [12]. However, the mechanisms regulating blood vessel growth during hypertrophy remain incompletely understood.

The nuclear lysine acetyltransferase (KAT) p300 is a nodal, dose-dependent regulator of the hypertrophic response to pressure overload [13]. In murine models, heterozygous deletion of p300 impairs the development of hypertrophy in response to pressure overload, while modest (2x) myocardial overexpression of p300 reproduces molecular and histopathologic phenomena associated with early, adaptive hypertrophy [13]. Here we show that p300-driven early adaptive hypertrophy is accompanied by a marked compensatory angiogenesis, associated with upregulation of multiple angiogenesis-regulating genes. Importantly, this positive effect is counterbalanced by upregulation of members of the anti-angiogenic miR17∼92 cluster [14], [15]. One member of this cluster, miR-20a, specifically targets p300 and negatively regulates the p300-dependent angiogenic transcription program, and angiogenic differentiation of cardiac progenitor cells. We conclude that the extent of myocardial angiogenesis is determined by a tight balance between competing pro- and anti-angiogenic factors during p300-driven adaptive responses.

## Experimental Procedures

### Materials and Reagents

Adenovirus vectors encoding p300 or a blank pcDNA3.1 vector were purchased from Cell Biolabs (San Diego, California). Lentiviral vectors encoding miR-20a or a control sequence within a common miR-30 backbone have been previously described [16]. Antibodies against p300, HDAC9, IgG and GAPDH were from Santa Cruz Biotechnology (Santa Cruz, CA); anti-GATA4 from Abcam PLC; anti-sarcomeric myosin heavy chain (MF20) was from the Hybridoma Bank (Iowa City, Iowa), anti-Vegfα from Abcam (Cambridge, MA); pan-alpha-actinin from Sigma-Aldrich; and anti-Hif1a from Millipore (Billerica, MA). The Amersham ECL Western detection system was obtained from GE Healthcare Bio-Sciences (Piscataway, NJ). The MirVana PARIS kit (Applied Biosystems/Ambion, Austin, TX) was used for microRNA extraction. Reagents for realtime quantitative PCR were obtained from Applied Biosystems, Foster City, CA, including TaqMan 5′ nuclease, Universal PCR Master Mix, MultiScribe reverse transcriptase and transcript-specific probes. Luciferase vectors encoding p300 and control 3′UTR sequences were obtained from GeneCopoeia, Rockville, MD.

### Mouse Models

Mice expressing α-MHC–driven transgenes encoding a full length human EP300 cDNA have been previously described [13]. All animals were treated in accordance with the Guide for the Care and Use of Laboratory Animals (National Institutes of Health), and according to protocols approved by the University of Miami Animal Care and Use Committee [13].

### *In vivo* Transduction of miR-20a

Newborn (5 day) p300tg pups (BS line [13]) received 8×10^8^ viral particles of miR-20a or control sequence (ct-miR) [16] via external jugular vein injection using a previously described method [17], with minor modifications [18]. In brief, 5-days old p300tg pups were placed on a transilluminator and the external jugular vein was injected with 10 µl of 8×10^8^ viral particles encoding miR-20a or ct-miR. Injected pups were returned to their mothers until weaning at 3 weeks of age.

### Microscopy and Stereology

Wild type and p300tg (BS line [13]) littermates were anesthetized and sacrificed by cervical dislocation. Total heart weight and left ventricle, liver and kidney weight and right tibia length were determined. Tissues were fixed in 4% paraformaldehyde in PBS and embedded in paraffin for hematoxylin and eosin (HE) and Picrosirius red staining. Stereological analysis was performed as previously described [13], [19]. For immunofluorescence imaging, cardiac myocytes cultured on Poly-L-Lysine-coated coverslips were washed and fixed in 4% paraformaldehyde × 15 min and then permeabilized with 0.2% Triton X-100 × 5 min. After 30 min blocking with goat serum, cells were incubated with primary and secondary antibodies, washed 3 times with PBS, and mounted using Anti-Fade DAPI (Invitrogen). Fluorescent images were acquired on Olympus FV1000 confocal microscope and processed using FluoView10ASW (Olympus).

### Western Blots

Immunoblotting was performed using standard methods. In brief, miRvana Paris kit (ABI/Ambion) was used for cell lysis or tissue homogenization. The lysate fractions were subjected to SDS-PAGE and transferred to nitrocellulose. Membranes were blocked for 1 hour at room temperature in 5% nonfat milk in a buffer containing Tris 20 mM, sodium chloride 137 mM, 0.5% Tween-20 pH 7.5 (TBS-T) and then incubated for 1 hour at room temperature with primary antibodies: followed by incubation for 45 minutes with horseradish peroxidase-conjugated secondary antibody. Proteins were imaged by chemiluminescence and bands were digitized and analyzed using Image J software (NIH, Bethesda, Maryland).

### Microarray Analysis

RNA was prepared from left ventricles of wild type and p300tg littermate mice (BS line [13]) at 13 weeks of age, using Trizol according the manufacturer’s instructions. The samples were DNAse digested and low-molecular weight (LMW) RNA was isolated by ultrafiltration through YM-100 columns (Millipore) and subsequent purification using the RNeasy MinElute Clean-Up Kit (Qiagen). The LMW RNA samples were 3′-end labeled with Oyster-550 fluorescent dye using the Flash Tag RNA labeling Kit (Genisphere). Labeled LMW RNA samples were hybridized to ORB version 1 spotted oligonucleotide MicroRNA Microarrays (Sanger version 9.0 microRNA database; Ocean Ridge Biosciences Jupiter, Florida) according to conditions recommended in the Flash Tag RNA labeling Kit manual. Microarrays were scanned on an Axon Genepix 4000B scanner, and data was extracted from images using GenePix V4.1 software. Data have been deposited in NCBI’s Gene Expression Omnibus [20] and are accessible through GEO Series accession number GSE50843 (http://www.ncbi.nlm.nih.gov/geo/query/acc.cgi?acc=GSE50843).

### Quantitative RT-PCR

Realtime PCR was carried out as previously described on an ABI 7900HT thermocycler [13].

### Chromatin Immunoprecipitation (ChIP)

ChIP was carried out as described in [21], with minor modifications. Briefly, left ventricles were dissected from hearts of 1 month old wt and p300tg littermates, finely minced, washed in 2× volume of 1× PBS, and fixed in 1/10 volume of fresh 11% formaldehyde for 10 min at RT. Formaldehyde was then quenched by adding 1/20 volume of 2.5 M glycine and immediately placing cells on ice. Cells were then homogenized by a mechanical homogenizer and passed through 100-mm nylon cell strainer. Cells were pooled and spun at 1,100 *g* for 5 min at 4°C, washed and resuspended in PBS and sonicated using a Microson ultrasonic cell disruptor to shear DNA to an average length of 500–700 bp. 1% Triton X-100 was added to sonicated lysate which was then centrifuged at 20,000 *g* for 10 min at 4°C to pellet debris. Input chromatin concentrations for each sample was normalized using an ND-1000 spectrometer (Nanodrop Technologies, Wilmington, DE). 100 ml antibody/magnetic bead mix was added to cell lysates and samples were incubated overnight on a rotator at 4°C. Materials were eluted from beads in buffer consisting of 50 mM Tris-HCl, pH 8.0, 10 mM EDTA, and 1% SDS at 65°C, and the supernatant was transferred to fresh tubes. Eluted DNA was reverse-crosslinked by heating at 65°C for a minimum of 6 hours. Finally, DNA was purified by phenol-chloroform extraction, dissolved in distilled H_2_O and amplified by RT-PCR. ChIP-qPCR primers used to amplify the 500 nt upstream enhancer of Egln3 were (F) CTTCTCAGGGGATACACACACCAG; (R) TGACCCCAACGTCATGGT. Primers used to amplify the Angpt2 enhancer 500 nt downstream of the transcriptional start site were (F) TGAGAGTGCGACAGAGCAGTA; (R) GGAAGTTGAGTCCAGAGAGG. Data are presented as a ratio of the cycle thresholds obtained using ΔΔCt method in the immunoprecipitated sample (IP) and input lanes. All amplicons were visualized on 2% agarose gels containing 0.4 mg/ml ethidium bromide. Each data point was obtained by pooling left ventricular muscle from 6–12 hearts per condition.

### Adenoviral Transduction of p300 in Primary Cardiac Myocytes

Cardiac myocytes were isolated from d1–3 neonatal Sprague-Dawley rat pups as previously described [22] and plated in 60 mm culture dishes in MEM +5% FBS. On the next day, cells were transduced using adenovirus encoding p300 or a blank pcDNA3.1 vector, and maintained thereafter in a defined serum-free medium as previously described [23]. Cells were harvested 48 hours after transduction and RNA was isolated as described above.

### Cardiac Progenitor Cell (CPC) Culture and Proliferation Assay

CPCs were isolated by Sca-1 affinity and cloned by serial dilution from adult mouse ventricular muscle as previously described [24]. CPCs were transduced with miR-20a or scrambled control lentivirus [16]. After 48 hours, cells were collected for RNA and protein work as described above. For proliferation assays, untransfected or lentivirus vector-transduced CPCs were plated in 60 mm tissue culture dishes at a density of 5×10^4^ per plate and counted daily for 6 days on a Coulter Counter (Beckman).

### Luciferase 3′UTR Assay

Luciferase vectors containing either full-length mouse p300 (NM_177821.6) 3′ UTR or a control 3′ sequence were transfected into 293T cells stably overexpressing miR-20a or a scrambled control microRNA using Lipofectamine 2000 (Invitrogen). 48 hours post-transfection, cell extracts were assayed for luciferase activity using the Luc-Pair miR luciferase assay kit (Genecoepia). Relative reporter activities were finally expressed as luminescence units normalized to Renilla luciferase activity in the same extracts. The miR-20a binding site in the p300 3′UTR was mutated using a site-directed mutagenesis kit (Stratagene). Primers for mutagenesis were: TTTCCTCAGCCCGTGAAAGAATTTAAATAAAAAAAAAGAACATTTC (F); TTATTTAAATTCTTTCACGGGCTGAGGAAAATAGGGAAAAATGTC (R).

### Statistical Analysis

For qRT-PCR mRNA analyses and all other statistical tests, differences between groups were assessed by one-way ANOVA. In all cases, the acceptance level of significance was p<0.05 using a two-tailed distribution. All transgenic mouse measurements were made in comparison to wt littermates.

## Results

### p300 Induces Blood Vessel Growth in the Myocardium during Hypertrophy

**Figure 1:**
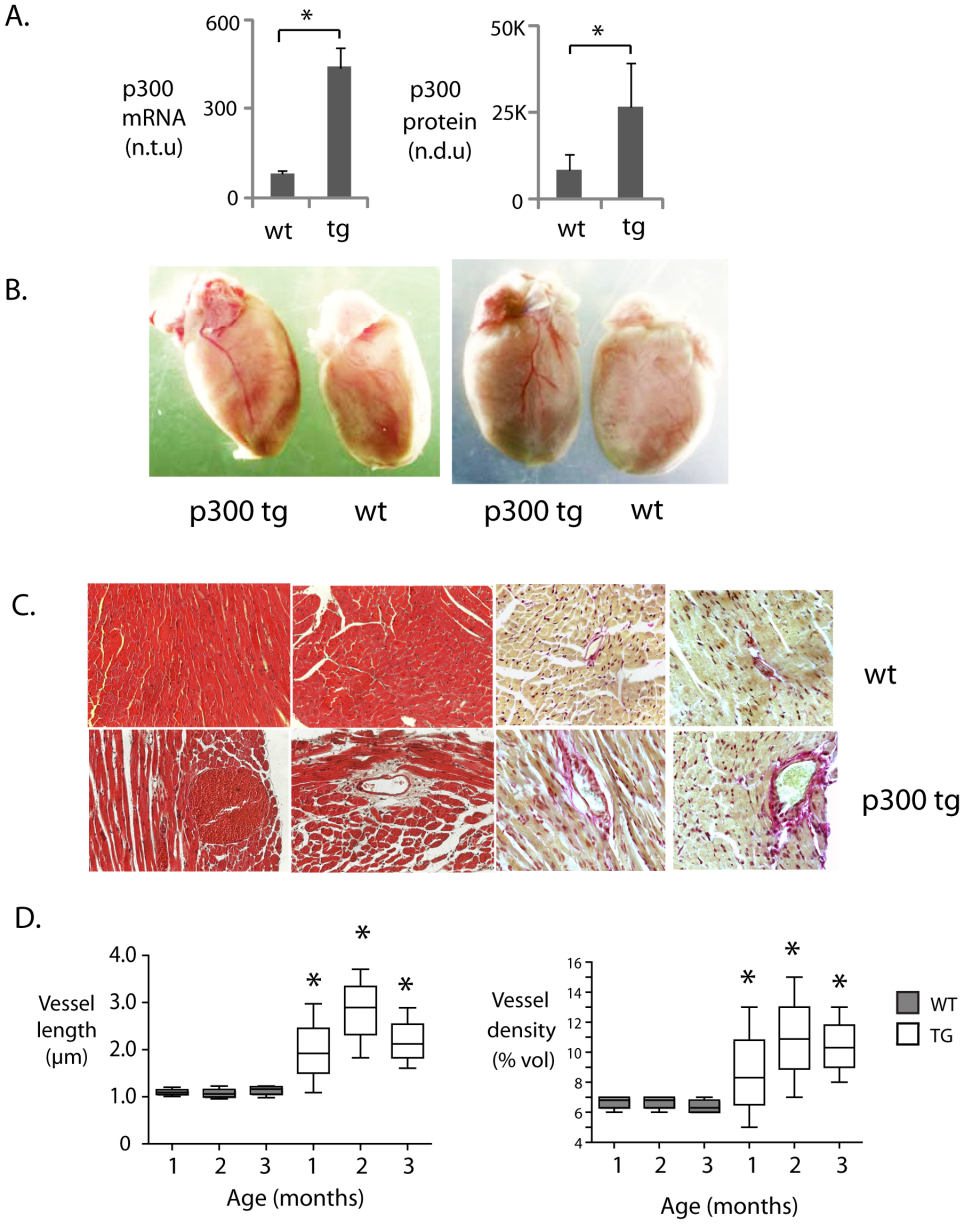
Cardiac-specific overexpression of p300 induces compensatory blood vessel growth. A. Alpha-MHC promoter-driven transgenic upregulation of p300. p300 mRNA and protein are upregulated at 1 month of age in p300tg relative to wt left ventricular muscle (LV). n = 4 per group; * = p<0.05; n.t.u = normalized transcript units; n.d.u = normalized densitometry units. **B. Enlargement of epicardial coronary vessels in p300 tg hearts.** Anterior (left) and posterior (right) views of p300tg (TG) and wild type (WT) littermate hearts at 6 months of age. **C. Increased blood vessel density in p300tg relative to WT myocardium.** Hematoxylin and eosin staining (left) and picrosirius red (right) of myocardial tissue from 3 month wt (top row) and p300 tg (bottom row) littermate hearts. Picrosirius staining is shown in brightfield and epi fluorescence. Original magnification = 10x. **D. Quantitatitve morphometry** of images as in C. Left: median vessel length in wt (shaded) and tg (clear) at the indicated ages. Right: vessel density as a percentage of tissue volume (% vol). n = 5 per group, * = p<0.001 at all time points for comparison between wt and p300 tg mice.

Mice expressing a myocardial-targeted human EP300 transgene displayed 2.5x increases in p300 mRNA and protein (Figure 1A), accompanied by a significant increase in absolute and normalized heart weight relative to their wt littermates (Table 1). Interestingly, p300tg hearts also showed enhanced vascularity, with prominent epicardial and intramyocardial coronary blood vessels, surrounded by perivascular elastin and collagen (Figures 1B, 1C). Quantitative stereological analysis confirmed significant increased volume density and length of intramyocardial blood vessels (Figure 1D).

**Table 1:** Organ weights in p300tg and wild type littermate mice.

	Wild type 1 month	p300 Transgenic 1 month	Wild Type 5 months	p300 Transgenic 5 months
**Body weight (g)**	17.4±0.84	16.3±1.23	28.4±4.43	26.5±1.46
**Heart weight (mg)**	80.1±2.68	102.3±7.10*	118.6±7.29	195.8±24.58*
**HW/BW**	4.8±0.32	6.3±0.14*	4.7±0.89	7.5±1.22*
**LV (mg)**	50.3±4.35	55.6±1.53	85.1±7.64	129.1±17.02
**Tibia length (mm)**	15.0±1.05	14.6±1.17	20.0±0.00	20.3±0.25
**HW/TL**	5.3±0.26	7.1±0.68*	5.9±0.36	9.7±1.24*
**Lung weight (mg)**	149.5±20.02	128.8±10.65	163.9±8.44	258.3±46.50
**Liver weight (mg)**	841.1±89.6	969.8±263.2	988.9±108.4	1244.0±140.7

Organ weights and tibia length were measured in 1 month and 5 month old p300 tg and wild type littermates (4 female mice per group. *p<0.05. Significant increases in heart weight, heart weight/body weight, and heart weight/tibia length ratios are seen in p300tg mice, as previously reported (Wei *et al*., 2008).

**Figure 2:**
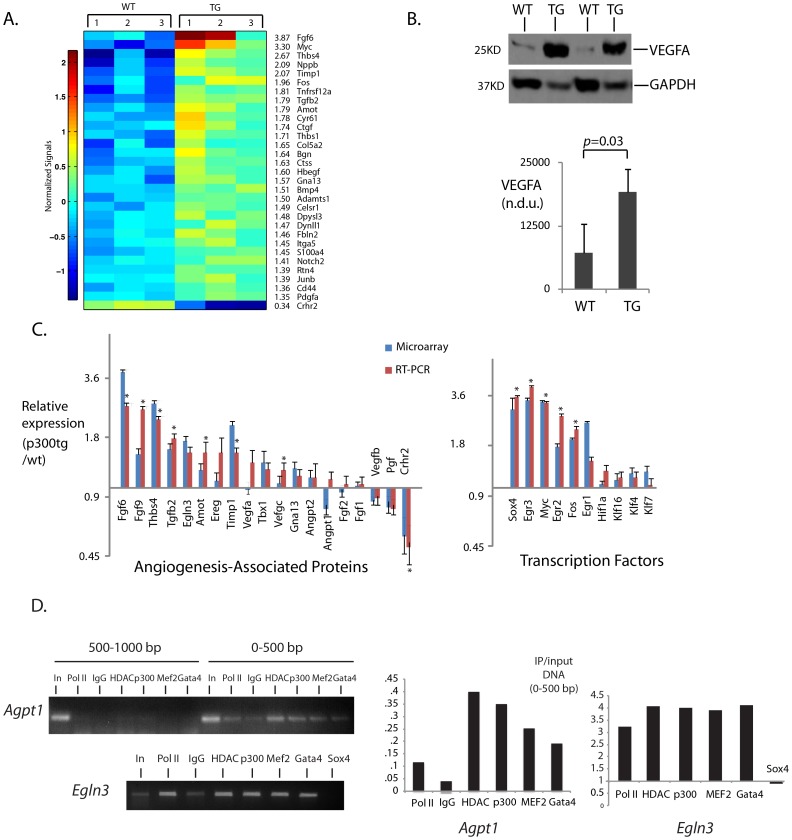
p300 activates an angiogenic transcriptional program. **A. Heatmap.** 23 differentially expressed angiogenic genes identified by genome-wide transcription analysis of left ventricular muscle from p300tg and wt littermates at 1 month. Shown are transcript profiles from each of 3 animals per genotype, cutoff > = 1.4 fold, p<0.02). Colorbar represents normalized intensities. Shown to the right of the heatmap are the relative changes of transgenic to wild type. **B.** **Comparative VEGFA protein levels in p300tg and wt littermate mice at 3 months.** (Above) Representative Western blot. Below, quantitation of 3–4 separate determinations per group. **C. qPCR validation of microarray-identified genes.** N = 3 animals per genotype. *p<0.05. **D. Binding of p300 to enhancers in Angpt1 and Egln3 genes.** Myocardium from 1 month-old C57Bl/6 mice was subjected to ChIP analysis using the antibodies shown. HDAC = HDAC-9. Pol II = RNA polymerase II. Data were obtained from 6–12 pooled left ventricles per condition.

To investigate the mechanism of this enhanced blood vessel formation, we performed microarray analysis comparing p300tg and wt littermate myocardial gene expression between 1 month of age revealed broad induction of angiogenesis-associated genes (cutoffs ≥1.4x, p<0.02, Figure 2A and Table 2). Selected genes were subsequently confirmed by RT-PCR assay, along with an additional 4 genes that did not pass the original microarray filter (Egr3, Egr2, Fgf9, and Vegfc, Figure 2C). Consistent with this, myocardial VegfA protein levels were also elevated relative to wt littermates (2.6±0.47 fold, p<0.05; Figure 2B). Chromatin immunoprecipitation assay confirmed p300 binding to potential regulatory sites in a subset of these genes, including Angpt1 and Egln3 (Figure 2D).

**Table 2:** Angiogenic genes differentially expressed in 1-month old p300tg hearts.

Gene Symbol	Gene Name
**Fgf6**	fibroblast growth factor 6
**Myc**	myelocytomatosis oncogene
**Thbs4**	thrombospondin 4
**Nppb**	natriuretic peptide precursor type B
**Timp1**	tissue inhibitor of metalloproteinase 1
**Fos**	FBJ osteosarcoma oncogene
**Tnfrsf12a**	tumor necrosis factor receptor superfamily, member 12a
**Tgfb2**	transforming growth factor, beta 2
**Amot**	angiomotin
**Cyr61**	cysteine rich protein 61
**Ctgf**	connective tissue growth factor
**Thbs1**	thrombospondin 1
**Col5a2**	procollagen, type V, alpha 2
**Bgn**	biglycan
**Ctss**	cathepsin S
**Hbegf**	heparin-binding EGF-like growth factor
**Gna13**	guanine nucleotide binding protein, alpha 13
**Bmp4**	bone morphogenetic protein 4
**Adamts1**	a disintegrin-like and metallopeptidase
**Celsr1**	cadherin EGF LAG seven-pass G-type receptor 1
**Dpysl3**	dihydropyrimidinase-like 3
**Dynll1**	dynein light chain LC8-type 1
**Fbln2**	fibulin 2
**Itga5**	integrin alpha 5
**S100a4**	S100 calcium binding protein A4
**Notch2**	Notch gene homolog 2 (Drosophila)
**Rtn4**	reticulon 4
**Junb**	Jun-B oncogene
**Cd44**	CD44 antigen
**Pdgfa**	platelet derived growth factor, alpha
**Crhr2**	corticotropin releasing hormone receptor 2

Microarray was performed on LV myocardial tissue from 3 mice per genotype, one array per mouse. Data were analyzed using GeneSpring software and filtered by p value (≤0.05) and extent of differential expression (>1.5x up or <0.66x reduced). Gene list corresponds to heatmap in Figure 2A.

### Inverse Expression of miR-17∼92 and VegfA

**Figure 3:**
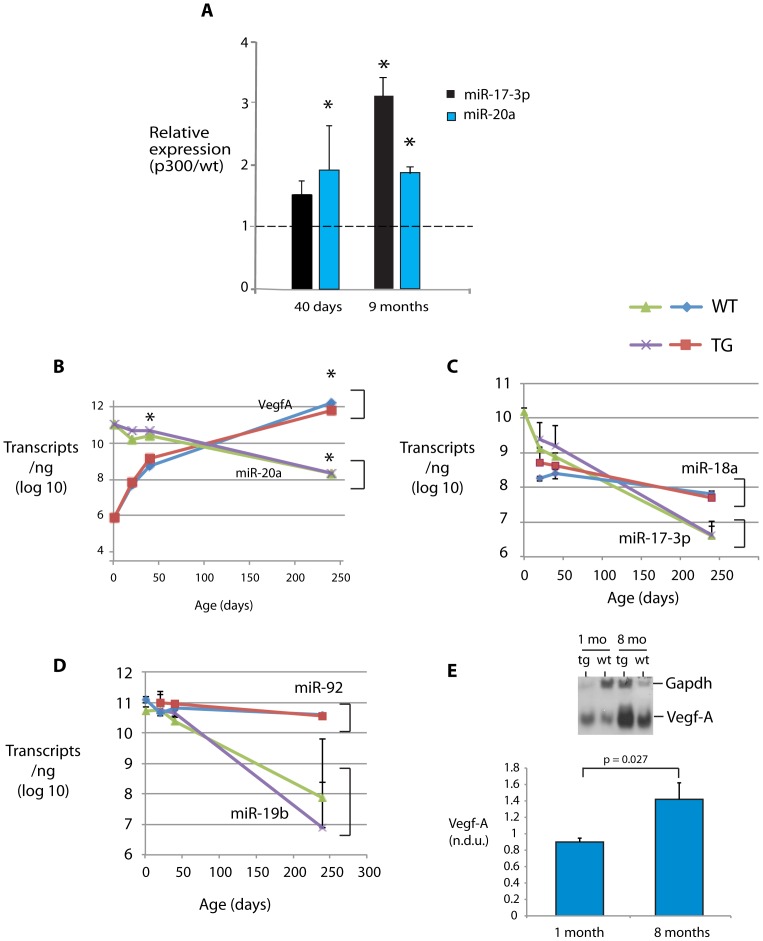
Reciprocal regulation of myocardial VegfA and miR-17∼92 with age. VegfA and mature miR transcript levels were determined by quantitative PCR in mice at the indicated time points. **A. Relative upregulation of miR-17-3p and miR-20a in p300 transgenic mice.** The ratios of p300 to wt levels for each transcript are shown. * p<0.05. **B. Time dependent inverse regulation of VegfA and miR-20a.** For B-D, absolute transcript levels in log 10 are shown. * p<0.05 for comparison between wt and p300. **C. Age-dependent downregulation of miR 17-3p and -18a. D. Age-dependent decline in miR-19b and -92.** **E. Increasing myocardial VegfA protein content between 1 and 8 months.** Above: representative Western blot showing comparative VegfA levels in wt and p300tg mice at 1 and 8 months of age, with Gapdh as loading control. Below: Quantitation of normalized Vegf protein expression in wt mice at 1 (n = 3) and 8 (n = 2) months.

We next asked whether microRNAs might be involved in the regulation of myocardial angiogenesis by p300. A microarray screen of microRNA expression comparing 1 month old p300tg with their wt littermates revealed a number of microRNAs with differential expression. Some of these have been reported previously [18]. Two members of the miR-17∼92 cluster, miR-17-3P and miR-20a, were upregulated in p300tg relative to WT myocardium in both young (1 month) and adult (9 month) mice (Figure 3A). However, the absolute levels of these and 2 other miRs in this cluster declined steadily with age in both p300tg mice and their WT littermates (Figure 3B–D). This decline in miR-17∼92 cluster expression was accompanied by a steady rise in expression of VefgA at both mRNA (Figure 3B) and protein (Figure 3E) levels, suggesting that members of this cluster play a repressive role in myocardial vascular growth.

### Co-regulatory Expression of p300 and miR-17∼92

**Figure 4:**
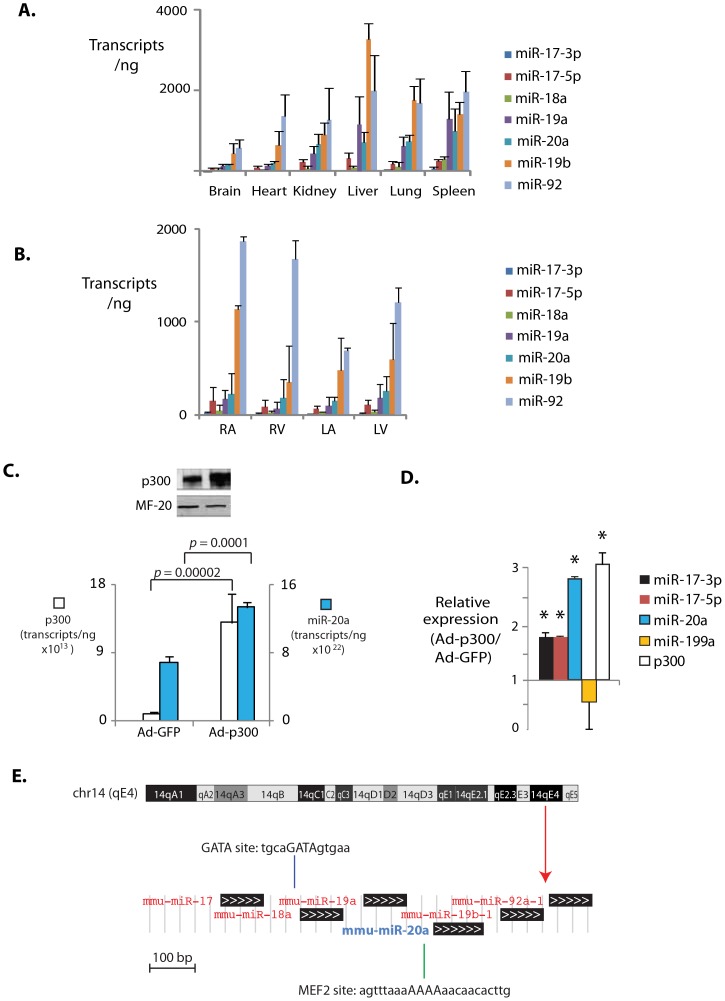
Impact of tissue type, p300 content and genomic context on miR-17∼92 cluster expression. **A. Comparative expression of 7 members of the miR-17∼92 cluster in normal tissues.** Tissues were harvested from mice at 3 months of age and RNA was quantitated for the indicated microRNAs. **B. Comparative miR-17∼92 cluster expression in normal murine heart.** LA: left atrium. RA: right atrium. LV: left ventricle. RV: right ventricle. Transcript levels expressed per ng total RNA. Note relatively higher expression of 3′ members of the cluster, miR-20a, miR-19b, and miR-92. **C. Gain of p300 induces miR-20a.** Above: p300 immunoblot of cardiac myocyte lysates 48 h after transduction with Adp300 or a control virus (AdGFP). (below) p300 and miR-20a transcript levels are both increased following transduction of Adp300 but not AdGFP. **D. Gain of p300 induces multiple members of miR-17∼92 cluster.** Relative expression of miR-17∼92 cluster members and an unrelated microRNA, miR-199, in Adp300- vs AdGFP-expressing cardiac myocytes. For A through C, N = 4 per group. * = p<0.05. **E. Genomic structure of miR17∼92 cluster.** Red arrow = cluster position on murine chromosome 14. Shown are locations and sequences of canonical MEF2 and GATA binding sites upstream of miR-20a.

All members of the miR-17∼92 cluster were expressed in all tissues examined (Figure 4A) and in all 4 chambers of the heart (Figure 4B) consistent with previous reports [25]. The absolute and relative expression of individual miRs were similar in most tissues, with greater expression of the 3′ end of the cistron (Figure 4AB). Adenovirus-mediated p300 expression significantly increased levels of all cluster members including miR-20a (1.95-fold ±0.03, p<0.0001, Figures 4C and D), while expression of an unrelated miRNA, miR-199, was unchanged (Figure 4D), confirming that p300 gain can positively regulate expression of this anti-angiogenic microRNA cluster. Although we were unable to confirm direct regulation by p300, MiR-17∼92 harbors conserved MEF2 and GATA binding sites within 500 bp downstream of the transcriptional start site that may reflect the presence of a p300-responsive enhancer (Figure 4E).

### miR-20a Represses the Angiogenic Gene Expression Program in CPCs

**Figure 5:**
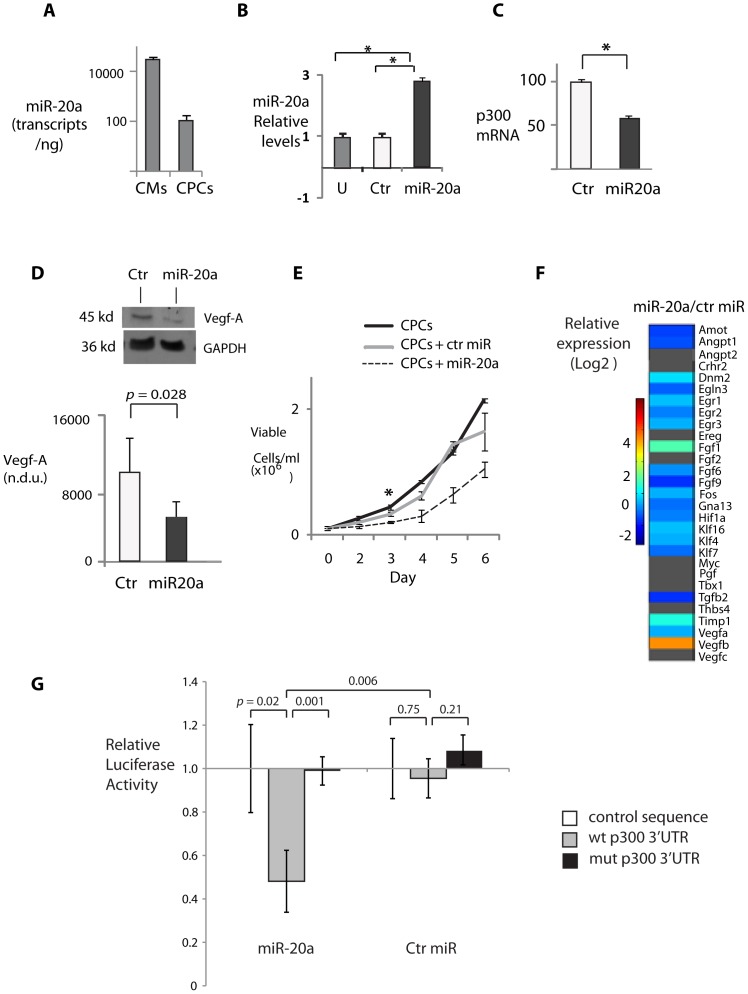
MiR-20a targets p300 and p300-regulated angiogenic genes. **A. Low expression of miR-20a levels in cardiac progenitor cells.** Absolute MiR-20a transcript levels in CPCs vs. neonatal cardiomyocytes were measured by qPCR in equivalent 10 ng total RNA samples. **B.** **Successful expression of miR20a in CPCs following lentiviral transduction.** Ordinate is in log_10_ scale. U = uninfected. Ct-miR: scrambled sequence. M = miR 20a sequence. ** = p<0.001. *p<0.05. **C. miR-20a reduces endogenous p300 transcript levels in CPCs. D.** **MiR-20a reduces Vegf-A protein in CPCs.** (Top) representative Western blot. (Bottom) Summary of 3 independent experiments**. E. Overexpression of miR-20a slows proliferation of cardiac progenitor cells (CPCs).** CPC clones stably expressing GFP and miR-20a or a scrambled sequence (ct-miR) were plated and maintained at 40–60% confluency by serial passage. Cell counts were determined as described in Experimental Procedures. **F.** **Mir-20a overexpression represses p300-induced angiogenic genes in CPCs.** Angiogenic genes identified as p300-regulated in the myocardial expression profile from Figure 2A were assayed by QPCR in CPCs expressing miR-20a or ct-miR. Data represent the average of 3 biological replicates per transcript. Color bar denotes relative expression levels. Grey = expression below detection threshold **G. MiR-20a directly targets EP300 3′UTR.** See Experimental Procedures for miR-20a binding site sequence and mutant sequence.

The 3′UTR regions of several p300-upregulated angiogenic genes, including HIF1a [26], [27] as well as p300 itself, contain the shared target site for miR-17-5p and 20a. To determine whether miR-20a could provide a counter-regulatory signal during p300-induced angiogenic transcription, we transduced miR-20a into previously described clonal murine c-kit+ cardiac progenitor cells (CPCs) [24]. These CPCs have high angiogenic differentiation potential and express endogenous miR-20a at very low levels relative to cardiomyocytes (Figure 5A). Lentiviral overexpression of miR-20a, but not a scrambled sequence [16] increased miR-20a levels by more than 600-fold (Figure 5B) from a negligible endogenous level; this was sufficient to reduce endogenous CPC p300 mRNA levels by more than 40% (Figure 5C) and VegfA protein levels by ∼50% (Figure 5D), associated with a significant reduction in CPC proliferation (Figure 5E). In fact, miR-20a overexpression significantly repressed most of the angiogenic genes that were differentially upregulated in p300tg hearts (Figure 5F; compare Figure 2A). Transduction of miR-20a, but not a control miR, repressed expression of a luciferase construct containing the p300 3′UTR miR17-5p/miR-20a binding site (Figure 5G, left); mutagenesis of this site eliminated miR-20a repression (Figure 5G, right). Neither vector inhibited a control sequence lacking the miR-20a binding site. These experiments confirm that p300 is a direct target of miR-20a, likely affecting the stability of p300 mRNA, and that angiogenic transcription downstream of p300 is also repressed by miR-20a.

### MiR-20a Alters Actinin Content in Cardiomyocytes

**Figure 6:**
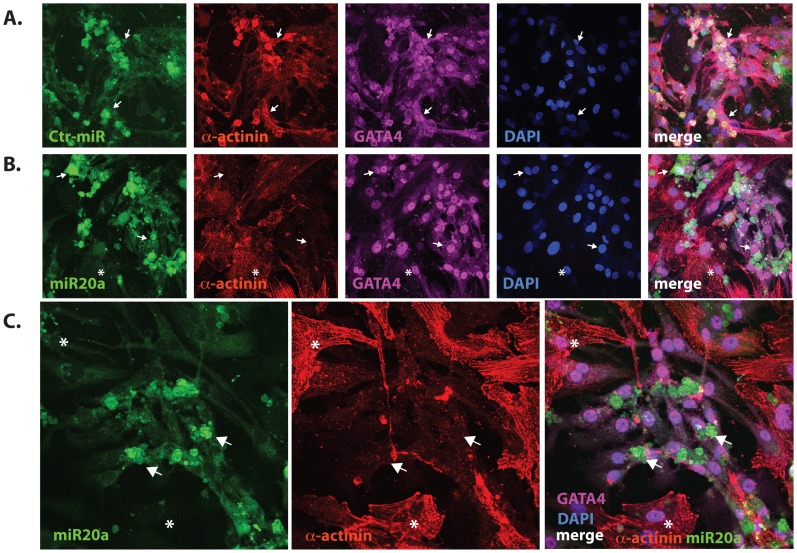
MiR-20a reduces actinin content in cardiomyocytes. Lentiviral-transduced cardiomyocytes expressing a control sequence (**A**, ct-miR) or miR-20a (**B** and **C**) together with EGFP (green) were visualized with anti-GATA-4 (magenta) and pan-alpha-actinin (red) antibodies and nuclear DNA was stained with DAPI (blue)**.** Note that cells expressing the miR-20a-EGFP lentivirus (**B and C,** arrows) have greatly reduced staining for actinin, compared with adjacent non-transduced cells (**B and C,** asterixes), or cells taking up the scrambled sequence (compare EGFP+ cells in **A**). Original magnification = 32x.

In myocardial cells, transcription of several sarcomeric genes is regulated in a p300-dependent manner [23]. We asked whether the sarcomeric organization of neonatal rat cardiomyocytes could be impacted by increased levels of miR-20a. Lentiviral transduction of miR-20a, but not a control sequence, sharply reduced the number of visible sarcomeres containing alpha-actinin in cardiac myocytes (Figure 6). Since sarcomeric actinins are not predicted targets of miR-20a, this could be due to loss of p300 and previously demonstrated p300-dependent actinin transcription [28]. These data further support functional repression of p300 by miR-20a.

### MiR-20a Represses p300-dependent Genes in the Myocardium in vivo

**Figure 7:**
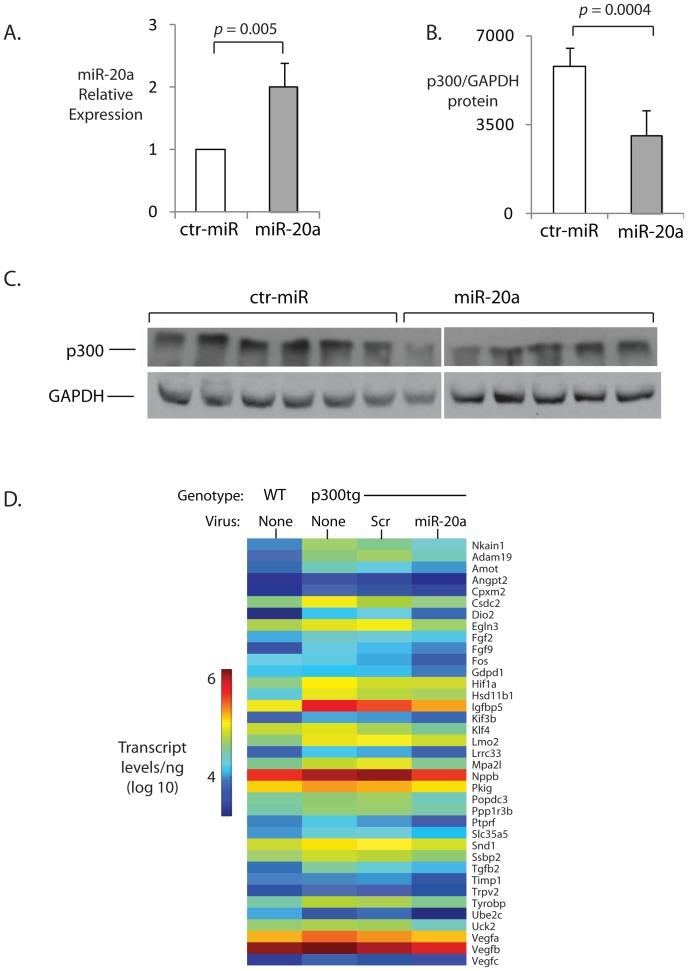
MiR-20a represses p300 and p300-driven angiogenic transcription *in vivo*. **A. Sustained *in vivo* transduction of miR20a.** 5-day old wt pups were transduced with the indicated lentiviruses and miR20a expression was detected by qPCR in left ventricular myocardial lysates at2 weeks. **B. Reduced p300 in miR-20a-transduced hearts.** p300 protein was quantitated in the same lysates in (A) by Western blot 3 months after transduction with miR-20a. n.d.u = normalized densitometry levels; * = p<0.05, n = 5. **C. Representative Western blot of data summarized in (B).** p300 and GAPDH bands were separately detected on 6% and 10% gels, respectively, with all 12 bands per protein quantified from a single autoradiogram. **D. miR-20a reverses myocardial p300-induced gene expression profile.** Absolute expression levels (log_10_) of p300-induced angiogenic genes in wt and p300tg mice with indicated treatment (n = 4–8/condition).

To determine whether miR-20a could similarly repress p300-dependent transcription in vivo, we transduced neonatal (d 1–3) p300tg mice with lentivirus encoding mature miR-20a or a scrambled sequence (ct-miR), and at 3 months of age, quantitated expression of 84 genes previously shown to be selectively upregulated by p300 *in vivo* [13]. No mortality or overt morbidity was observed with either vector. We have previously demonstrated long-lasting effects of microRNA transduction using this method [18]; consistent with this, miR-20a levels were significantly elevated for at least 2 weeks following injection (Figure 7A) following miR-20a but not ct-miR transduction. MiR-20a recipients had significantly reduced myocardial p300 protein (Figures 7B and C), together with significant reversal of the myocardial p300 gene expression signature (Figure 7D and Table 3) relative to control miR-transduced hearts.

**Table 3:** p300-induced genes repressed by miR-20a transduction *in vivo*.

GeneSymbol	foldchange	*p*-value	SD	Predicted Mir-20abinding site?
**Ereg**	0.25	0.0004	0.06	Yes
**Ptprf**	0.60	0.0008	0.27	Yes
**Adam19**	0.48	0.0016	0.10	No
**Ube2c**	0.22	0.0022	0.11	No
**Egln3**	0.43	0.0032	0.08	Yes
**Timp1**	0.38	0.0034	0.16	No
**Popdc3**	0.50	0.0036	0.11	Yes
**Uck2**	0.51	0.0040	0.09	No
**Ssbp2**	0.70	0.0059	0.16	Yes
**Cpxm2**	0.57	0.0077	0.13	No
**Slc35a5**	0.66	0.0089	0.14	No
**Trpv2**	0.59	0.0092	0.19	No
**Igfbp5**	0.61	0.0124	0.04	No
**Nppb**	0.34	0.0165	0.20	No
**Csdc2**	0.65	0.0175	0.17	No
**Vegfb**	0.66	0.0178	0.39	No
**Tyrobp**	0.61	0.0186	0.08	No
**Ppp1r3b**	0.62	0.0209	0.18	Yes
**Fgf2**	0.76	0.0210	0.16	No
**Snd1**	0.69	0.0217	0.12	No
**Kif3b**	0.68	0.0226	0.19	Yes
**Tgfb2**	0.59	0.0230	0.12	No
**Dio2**	0.43	0.0266	0.58	No
**Klf4**	0.62	0.0287	0.25	No
**Angpt2**	0.62	0.0296	0.27	Yes
**Fos**	0.53	0.0301	0.20	No
**Nkain1**	0.57	0.0303	0.18	No
**Lrrc33**	0.54	0.0319	0.14	No
**Pkig**	0.77	0.0401	0.14	No
**Gdpd1**	0.66	0.0414	0.26	No
**Amot**	0.62	0.0419	0.13	No
**Fgf9**	0.73	0.0429	0.10	No
**Gsta2**	0.21	0.0529	0.29	No
**Lmo2**	0.69	0.0531	0.13	No

A previously defined subset of 86 transcripts specifically upregulated in p300tg vs wt littermate hearts (15) was assessed by qPCR 3 months after transduction with miR-20a or a scrambled sequence. Significantly altered genes constituted more than a third of the total; almost all were repressed. N = 4–6 mice per group. MiR-20a binding sites predicted by TargetScan.

## Discussion

Acetyltransferase p300 is a nodal regulator of the myocardial stress response. We have previously shown that during mechanical stress, myocardial levels of p300 rise rapidly and that this rise in p300 is both necessary and sufficient for myocardial adaptive hypertrophy [13]. Here we show that p300 also drives a broad angiogenic transcription program that results in the development of abundant blood vessels, and at least initially offsetting the vascular attenuation that may occur as muscle mass increases [29]. Remarkably, rising p300 levels also lead to the upregulation of counter-regulatory microRNAs that repress both angiogenic transcription and expression of p300. We show that p300 is a novel target for a microRNA, miR-20a, that has been previously implicated in the negative regulation of angiogenesis through its ability to target VEGF and HIF-1 [26], [27]. Similar negative feedback loops have been described for miR20a, E2F and Myc [30], [31], [32], [33].

Our findings are consistent with the recently proposed role for microRNAs in imparting robustness to biological systems through both positive and negative autoregulatory loops [34]. Because of the tight dose-dependence of hypertrophy on p300 levels, small reductions in p300 content and function can substantially mitigate the adverse consequences of sustained stress-adaptive hypertrophic signaling [13], [35]. Thus, there are clear adaptive benefits of a negative feedback system that can fine-tune and balance the hypertrophic response, permitting long-term adaptation. In addition, miR-20a has been recently reported to reduce apoptosis following hypoxia-reoxygenation of cardiomyocytes [36], and overexpression of the miR-17∼92 cluster modestly improved cardiac function in a mouse model of myocardial infarction [37], suggesting that members of this cluster may have more general protective effects during oxidative or biomechanical stress. On the other hand, unbalanced upregulation of miR-20a in this system could lead to excessive attenuation of angiogenic transcription, and could in theory account for eventual failure of compensatory blood vessel growth [12], [13], [38].

Angiogenesis in the myocardium is thought to arise in part from the differentiation of resident pluripotent stem/precursor cells expressing the c-*kit* cell surface marker [39], [40]. Notably, we find that miR-20a prevents the expression of angiogenic genes not only in the intact myocardium, but also in c-kit+ cardiac progenitor cells [40]. Members of the miR-17∼92 cluster can retard endothelial sprouting in Matrigel [41]; in addition, loss of miR-17∼92 is seen in several forms of cancer [15], [42], [43]. Deletion of miR-17∼92 is embryonic lethal, due in part to defects in cardiac development [14], [44] which could arise from altered vasculogenic regulation. MiR-92, another member of the cluster, has been shown to regulate myocardial angiogenesis through targets distinct from those of miR20a, including integrin subunit alpha5 [14]. Our data add to an increasing number of microRNAs shown to regulate aspects of angiogenesis [45], [46] and cardiac hypertrophy [47], [48], [49].

**Figure 8:**
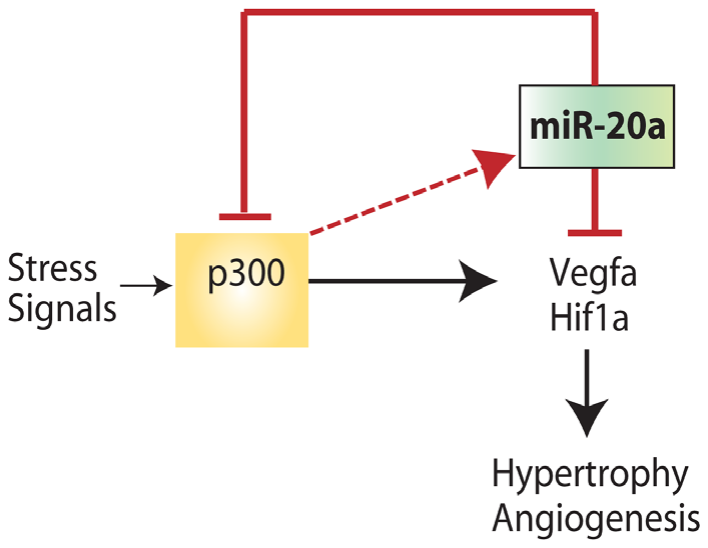
A model of the miR20-p300 feedback loop during hypertrophy. Stress signals acting on the myocardium induce p300 expression, which results in the activation of angiogenic and hypertrophic transcriptional programs leading to cardiac hypertrophy. p300 also induces miR-20a, which provides feedback inhibition of p300, reversing both the angiogenic and hypertrophic programs and providing counter-regulatory control of the hypertrophic stress response.

Our model is summarized in Figure 8. We postulate that the process leading to adaptive hypertrophy is initiated by extracellular stress signals, either mechanical or humoral, which upregulate myocardial p300. p300 then activates transcription of a set of genes required for hypertrophy and accompanying angiogenesis, likely through coactivation of apical effectors such as HIF1-alpha and VegfA. In parallel, p300 upregulation leads directly or indirectly to increased expression of miR-17∼92 cluster members that provides a brake on both processes and retards secondary maladaptive events. Whether compensatory and maladaptive hypertrophy are separate entities, or whether these processes lie on a continuum, remains unknown. It is plausible that the transition from one state to the other is governed by secondary molecular events that occur when the initial stress response is prolonged. Understanding the molecular events underlying both early and late stages of hypertrophy will be important to distinguishing between these possibilities. Our studies provide evidence that microRNAs, including those with important roles in angiogenesis, are important participants in the transition from hypertrophy to heart failure.
